# Bacteriophages Against *Aeromonas* Species in Aquaculture: An Integrative Review of Therapeutic Applications, Genomic Advances and Future Directions

**DOI:** 10.3390/v18080868

**Published:** 2026-08-09

**Authors:** İfakat Tülay Çağatay

**Affiliations:** Molecular Microbiology Laboratory, Department of Aquatic Basic Sciences, Faculty of Fisheries, Akdeniz University, 07050 Antalya, Türkiye; tulaycagatay@akdeniz.edu.tr

**Keywords:** phage therapy, lytic phages, phage cocktails, antimicrobial-resistant strains, fish disease management

## Abstract

Aquaculture has become the leading source of global aquatic food production, and its continued expansion has been accompanied by an increasing burden of bacterial diseases. *Aeromonas* spp. are among the most damaging bacterial pathogens in aquaculture and are recognised reservoirs of antimicrobial resistance within a One Health framework. Although bacteriophages have been investigated against numerous aquaculture pathogens, evidence relating to *Aeromonas*-targeting phages remains dispersed across studies. This integrative review synthesises research published between 1981 and 2026 on bacteriophages targeting *Aeromonas* spp. and examines the development of the field from early isolation studies to current therapeutic and translational applications. Studies were identified through structured searches of PubMed, Web of Science and Scopus and analysed for phage isolation, genomic characterisation, therapeutic efficacy, delivery strategies and phage-derived applications. Comparison of findings across studies reveals several consistent patterns. Phage cocktails generally provided broader antibacterial activity and reduced the emergence of antimicrobial-resistant strains compared with single-phage treatments. Preventive applications consistently produced higher survival rates than treatments applied after infection, while whole-genome sequencing has become a routine component of candidate phage evaluation. Recent studies have expanded the scope of phage-based interventions beyond intact virions to include phage-derived endolysins, oral delivery systems and phage lysate-based vaccine approaches. The evidence shows that bacteriophages can reduce mortality, suppress bacterial infections and improve survival across diverse aquaculture species affected by *Aeromonas* spp. Phage resistance, large-scale implementation and regulatory harmonisation remain important challenges, supporting further development of phage-based disease management in aquaculture.

## 1. Introduction

The global human population is projected to reach 10 billion by 2050, increasing food demand across all agricultural sectors [1,2,3]. Aquatic foods produced through capture fisheries and aquaculture have become central to food security and are known to supply approximately 15% of the world’s high-quality animal protein [4,5]. Aquaculture production is expected to rise by a further 10% by 2032, whereas capture fisheries remain largely stagnant and have been overtaken owing to declining wild stocks [6,7,8]. Meeting this demand has driven aquaculture toward increasingly intensive, high-density production systems [9,10].

Crowded rearing conditions and deteriorating water quality create a favourable environment for pathogen proliferation, and the globalisation of trade in live aquatic animals has facilitated the spread of pathogens across geographic boundaries. Environmental stressors such as hypoxia, temperature fluctuations and nutritional imbalances further compromise host immune defences, increasing susceptibility to infection [11]. These factors have made disease management one of the most critical challenges for sustainable aquaculture production [12].

Bacterial diseases stand out as the most economically destructive risk to aquaculture production. Globally, disease outbreaks cost the aquaculture industry an estimated USD 6 billion per year [13], a figure that captures direct production losses from mortality and morbidity as well as expenditure on antibiotics, vaccines and other control measures [14]. Large-scale vaccination programmes are now widely used in commercial aquaculture, reflecting the economic importance of bacterial disease control [15]. In major producing countries such as China, India and Vietnam, diseases account for over 30% of total production losses, and in intensive aquaculture nations such as Norway, Chile and Greece, the pressure of bacterial infections on production efficiency and economic sustainability is mounting steadily [16]. The range of bacterial pathogens involved is broad. Aeromonosis, Edwardsiellosis and Columnaris disease are among the most frequently reported and economically damaging bacterial diseases in freshwater aquaculture [17].

Streptococcal infections, Mycobacteriosis and Vibriosis add further pressure across diverse aquaculture systems, while *Streptococcus* spp. and *Mycobacterium* spp. drive high mortality and impaired growth in affected stocks [18]. In recent years, pathogens such as *Lactococcus garvieae* and *Edwardsiella piscicida* have added to the complexity, while *Aeromonas* spp. and *Vibrio* spp. remain responsible for some of the largest and most economically damaging disease outbreaks on record [11].

*Aeromonas* spp. are among the most important bacterial pathogens in aquaculture because of their widespread distribution, broad host range spanning farmed fish as well as crustaceans such as shrimp and crayfish, and extensive virulence repertoire. There are currently 35 formally described species within the genus *Aeromonas* [19], with *A. hydrophila*, *A. salmonicida* and *A. veronii* being the most frequently associated with disease in aquaculture [15,20,21,22,23,24]. These pathogens are capable of causing a wide range of clinical manifestations, including haemorrhagic septicemia, ulcerative lesions, fin rot and systemic infections, often resulting in high mortality rates. Their pathogenicity is driven by a diverse array of virulence factors, including aerolysins, haemolysins, enterotoxins, cytotoxins, gelatinase and secretion systems, which allow them to invade host tissues, evade immune responses and cause systemic infections [20,21,22]. *A. salmonicida* is the causative agent of furunculosis, a systemic disease responsible for high mortality in salmonid aquaculture worldwide [17]. *A. dhakensis* has recently been identified as a zoonotic pathogen of growing clinical concern, showing higher virulence than *A. hydrophila* in several comparative studies [25]. The best characterised species is *A. hydrophila*, a facultatively anaerobic freshwater bacterium that causes motile aeromonad septicaemia (MAS) in fish and is also recognised as an opportunistic zoonotic pathogen of humans. Together with *A. caviae* and *A. veronii* biovar *sobria*, it accounts for the majority of human clinical isolates, in which it causes acute gastroenteritis, wound and soft tissue infections that may progress to necrotising fasciitis, hepatobiliary infections and bacteraemia or septicaemia. These infections are frequently more severe in immunocompromised patients and are commonly linked to exposure to contaminated water or aquatic environments [20].

Antibiotics are the default response to the bacterial outbreaks encountered in aquaculture [26,27,28]. Global antibiotic use in aquaculture is expected to rise by another 33% between 2017 and 2030, with the sector already accounting for roughly 6% of total worldwide antibiotic consumption [11]. Antibiotics affect both pathogenic and non-pathogenic microbiota, can accumulate in aquatic organisms, and suppress the host immune response [29]. *Aeromonas* spp. have been shown to carry a diverse repertoire of antimicrobial resistance genes (ARGs) [30,31,32,33,34]. The dissemination of these ARGs from aquaculture environments into human clinical settings through food chains, water systems and direct contact represents a One Health concern, indicating that the consequences of antimicrobial misuse in fish farming extend beyond the farm [35,36].

Numerous non-antibiotic disease management strategies have been investigated to reduce antibiotic dependence in aquaculture. Vaccines, with licenced formulations available against bacterial diseases of farmed fish such as furunculosis (*Aeromonas salmonicida*), vibriosis (*Vibrio anguillarum*), yersiniosis (*Yersinia ruckeri*) and enteric septicaemia of catfish (*Edwardsiella ictaluri*) [37], prebiotics, probiotics, immunostimulants, bacteriocins, plant extracts and essential oils have been extensively investigated [24,38]. None has yet proven capable of fully replacing antibiotics in routine aquaculture practice [34]. Reducing antibiotic use while maintaining effective disease control remains a major objective in modern aquaculture health management [39,40,41].

Among the alternative approaches currently under investigation, bacteriophages (phages) have attracted considerable attention. These are viruses that specifically infect and replicate within bacteria, and their ability to selectively kill bacterial hosts has long made them attractive as targeted antibacterial agents, particularly against multidrug-resistant pathogens [23]. Bacteriophages follow two main life cycles: lytic phages replicate within the host and kill it by lysis, whereas temperate phages integrate into the host genome without immediate killing [42,43]. Lytic phages are therefore preferred for therapeutic use, as they selectively infect and lyse bacterial pathogens without disrupting the surrounding microbial community and without transferring resistance or virulence genes to the host [44,45,46,47,48,49,50]. Unlike antibiotics, phage therapy targets species-specific fish pathogens, does not accelerate the spread of resistance genes, and leaves no residues in aquatic organisms or their environment [51,52,53].

Research on phages targeting *Aeromonas* has expanded substantially over the past four decades, but the available literature remains scattered across studies focusing on different host species, phage characteristics and application strategies. Although several reviews have discussed phage therapy in aquaculture, most have adopted a broad multi-pathogen perspective, limiting detailed evaluation of developments specific to this genus. This review provides an integrative synthesis of research published between 1981 and 2026 on bacteriophages targeting *Aeromonas* spp. in aquaculture. It aims to evaluate the accumulated evidence across five dimensions: the host range and specificity of characterised phages, their therapeutic and prophylactic efficacy in aquaculture hosts, delivery and formulation strategies, genomic characterisation and safety, and progress toward practical and commercial application. By bringing these strands together within a single framework, it identifies consistent patterns, clarifies which stages are well supported by evidence and which remain underexplored, and highlights the key gaps and priorities that must be addressed to advance phage-based disease management against *Aeromonas* spp.

## 2. Materials and Methods

### 2.1. Review Design

**Figure 1:**
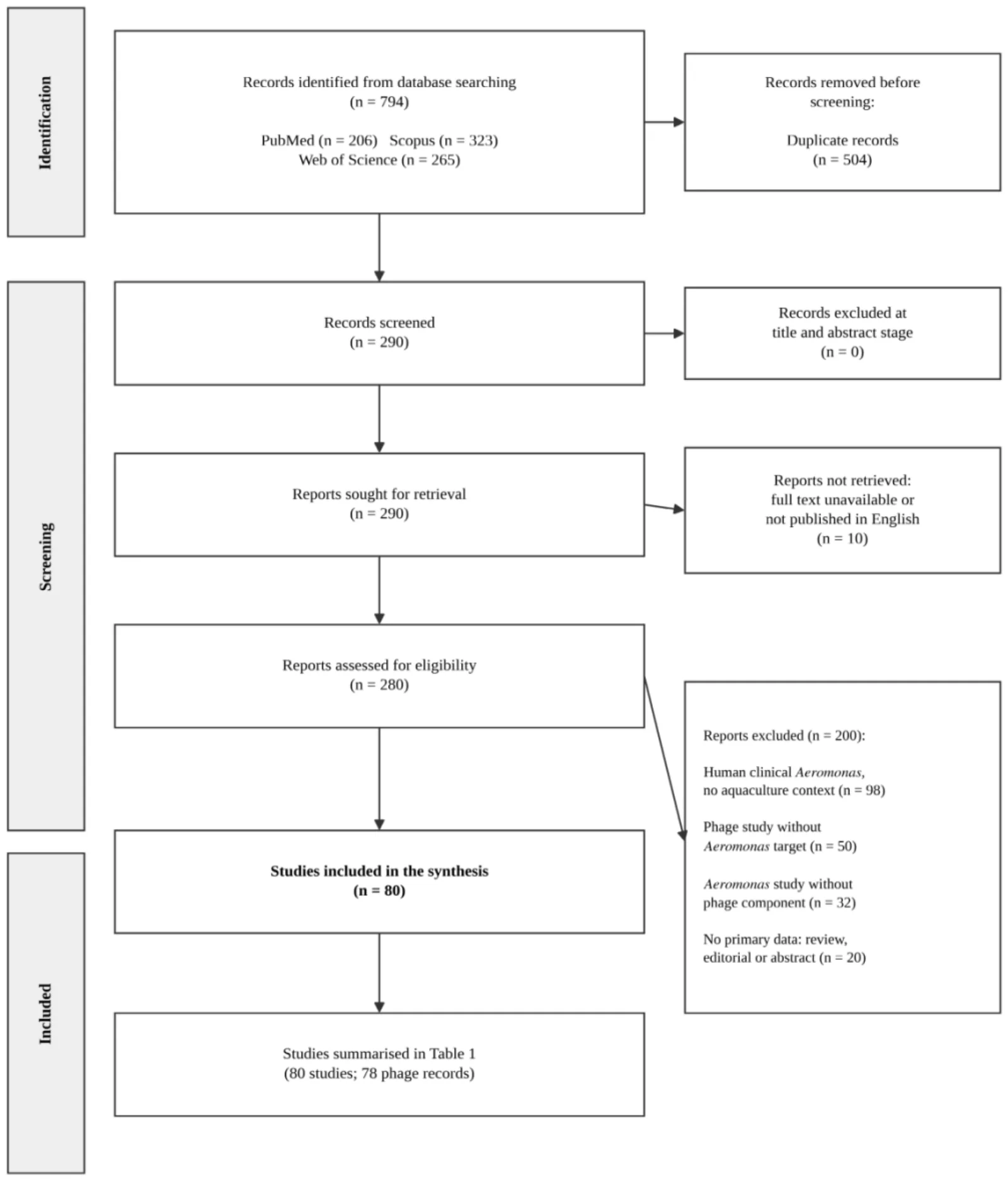
Flow diagram of study identification and selection, adapted from the PRISMA 2020 statement. This review was conducted as an integrative review; the PRISMA format is used for reporting transparency and does not indicate a registered systematic review protocol.

This study was conducted as an integrative review following the methodological framework described by Whittemore and Knafl [54], which allows experimental, genomic, and observational studies to be combined within a single synthesis. The review was not registered in a protocol database and was not conducted as a systematic review. To support transparency and reproducibility, the identification and selection of studies are reported in accordance with the reporting principles of the PRISMA 2020 statement, and the selection process is summarised in Figure 1.

### 2.2. Search Strategy

Three electronic databases were searched: PubMed, Scopus and Web of Science. The final search was performed on 5 April 2026 and covered publications from 1981 to 2026. The year 1981 was selected because it corresponds to the earliest documented application of bacteriophages against *Aeromonas* spp. in an aquaculture context. Searches were run separately for each target species. Search terms were combined with the Boolean operators AND and OR and were applied to title, abstract and keyword fields. The search string was structured as follows: (“bacteriophage” OR “phage”) AND (“*Aeromonas hydrophila*” OR “*Aeromonas salmonicida*” OR “*Aeromonas veronii*” OR “*Aeromonas caviae*” OR “*Aeromonas dhakensis*” OR “*Aeromonas sobria*” OR “*Aeromonas bivalvium*” OR “*Aeromonas schubertii*”).

Database-specific syntax was applied, using TITLE-ABS-KEY in Scopus, TS = in Web of Science and the [tiab] field tag in PubMed. Results were restricted to English-language publications. The number of records retrieved was 206 from PubMed, 323 from Scopus and 265 from Web of Science. Reference lists of the retrieved articles were screened manually as an additional check; this did not yield eligible studies beyond those already identified by the database searches.

### 2.3. Eligibility Criteria

Studies were eligible if they reported primary data on bacteriophages targeting *Aeromonas* spp. in aquaculture or fish disease systems and fell into one of three categories: (i) in vitro investigations reporting phage isolation, host range or lytic activity; (ii) in vivo studies evaluating therapeutic or prophylactic efficacy in aquaculture host species; and (iii) genomic studies characterising phage genome organisation or safety-related genetic content. Studies were excluded if they addressed *Aeromonas* infections in human clinical settings without an aquaculture context; investigated bacteriophages targeting bacterial genera other than *Aeromonas*; addressed *Aeromonas* without a bacteriophage component, for example taxonomic, virulence or resistance surveys; contained no primary data, such as reviews, editorials and conference abstracts; or were unavailable in full text or not published in English.

### 2.4. Study Selection

The database searches retrieved 794 records (PubMed, 206; Scopus, 323; Web of Science, 265). Because the searches were run separately for each target species, records retrieved by more than one species query were removed as duplicates, together with records retrieved by more than one database. This gave 504 duplicate records and 290 unique records for screening. Given the size of the resulting record set, title and abstract screening and full-text assessment were carried out in a single stage, and no records were excluded on the basis of title and abstract alone. Full texts could not be obtained for 10 records, which were either unavailable or not published in English, leaving 280 reports assessed for eligibility. Of these, 200 reports were excluded: 98 addressed human clinical *Aeromonas* infections without an aquaculture context, 50 examined bacteriophages targeting genera other than *Aeromonas*, 32 addressed *Aeromonas* without a bacteriophage component, and 20 contained no primary data. A total of 80 studies met the eligibility criteria and are summarised in Table 1, which presents 78 phage records because several studies reported more than one phage and two studies contributed to more than one record. Screening and selection were performed by a single reviewer. Additional literature on background topics, including aquaculture production trends, antimicrobial resistance, *Aeromonas* taxonomy and human infections, and bacteriophage applications in sectors other than aquaculture, was identified through supplementary searches and is cited in Section 1, Section 2, Section 3, Section 4 and Section 5. These sources were not part of the evidence base summarised in Table 1.

### 2.5. Data Extraction and Synthesis

The following variables were extracted from each included study: phage designation, target *Aeromonas* species and strain, host organism or environmental source, country of isolation, reported morphotype or taxonomic assignment, type of application, and the main reported outcome. Studies were then grouped into thematic categories to identify research trends, methodological developments and remaining gaps.

## 3. Disease Management in Aquaculture: From Antibiotics to Therapeutic Strategies

Antibiotics and other antimicrobials have long been used against bacterial disease outbreaks in aquaculture. Tetracyclines, quinolones, aminoglycosides, florfenicol and sulfonamides are among the most frequently used; however, approximately 70–80% of these are not absorbed by the target organism and instead accumulate in the surrounding water bodies, exerting selective pressure on the resident microbiota [17,24,28,136,137]. Resistance genes conferring resistance to β-lactams (β-lactamase genes, including the carbapenemase *bla*NDM), tetracyclines (*tetA*, *tetB*, *tetD*, *tetE* and *tet*(X4)), quinolones (*qnrA*, *qnrB* and *qnrS*), sulfonamides, amphenicols and colistin (*mcr-3*) are found together in isolates of fish, shrimp, crab and crustaceans in many countries and are regularly detected in pathogenic bacteria associated with aquaculture [40,138]. The dissemination of these resistance determinants through food chains and aquatic environments extends the consequences of antimicrobial use beyond aquaculture and reinforces the One Health dimension of antimicrobial resistance.

In response to these challenges, vaccination is still considered the most ecologically sustainable prophylactic strategy in intensive fish farming [139,140]; in parallel, probiotics and prebiotics are increasingly used as microbial management tools [141,142,143]. Other strategies include bacteriocins, a subclass of antimicrobial peptides primarily synthesised by lactic acid bacteria and *Bacillus* spp. [144]; plant compounds and essential oils such as thymol, carvacrol, cinnamaldehyde, eugenol, quercetin and resveratrol [38]; biosurfactants produced by certain bacteria and yeasts [9]; quorum sensing interference (QSI), which disarms pathogens without directly killing them [9]; and host-derived antimicrobial peptides (AMPs), cationic molecules found in fish epidermal mucus, gills and skin that disrupt bacterial cell membranes [24,145].

These alternative strategies share fundamental limitations: species specificity, sensitivity to environmental conditions, and the persistent challenge of achieving consistent dosing at commercial scale. No single alternative has consistently replaced antibiotic-based disease control across the diversity of aquaculture species and production systems [7,22,146,147]. Phage therapy differs from these approaches in several respects. Bacteriophages selectively eliminate target pathogens while preserving the surrounding microbiota, do not require prior immunological priming, and possess the capacity for in situ amplification in response to bacterial density [16,33,148,149].

## 4. Bacteriophage Applications

Bacteriophages are highly host-specific viruses that infect prokaryotic hosts [150,151,152,153]. Lytic phages destroy the host cell and release 50–200 progeny per lysed cell [154,155,156], whereas temperate phages integrate into the host chromosome and are therefore unsuitable for therapy, as they can mediate horizontal transfer of virulence and resistance determinants [157]. Interest in phage therapy has increased markedly in recent decades, driven by the global emergence of multidrug-resistant pathogens [158,159].

Phage taxonomy was reorganised in 2022. Previously, the International Committee on Taxonomy of Viruses (ICTV) placed tailed phages in the order *Caudovirales*, divided into three morphology-based families: *Myoviridae* (long contractile tails), *Siphoviridae* (long non-contractile tails) and *Podoviridae* (short tails) [52,160]. Because phylogenomic analyses showed these families to be polyphyletic, ICTV dissolved *Caudovirales* and replaced it with the class *Caudoviricetes*, which covers all tailed bacterial and archaeal viruses with icosahedral capsids and dsDNA genomes, and adopted binomial nomenclature [161]. The earlier morphological groupings remain in wide use in the applied literature. Throughout this review, phages reported by morphology alone are described using the informal terms myovirus, siphovirus and podovirus, whereas current ICTV taxa are given where genome-based assignments are available. Family names superseded by the 2022 reclassification are used only when the pre-2022 system itself is described.

Bacteriophages have been investigated across multiple sectors. In human medicine, patient-specific therapy has shown clinical efficacy against multidrug-resistant pathogens such as *Staphylococcus aureus*, *Acinetobacter baumannii* and *Mycobacterium abscessus* [162,163,164]. Veterinary applications target zoonotic pathogens such as *Escherichia coli*, *Salmonella*, *Campylobacter*, *S. aureus* and *Pseudomonas aeruginosa* [149,165]. The food industry uses commercially approved phage products, including ListShield, LISTEX P100 and SalmoFresh, which target *Listeria monocytogenes* and *Salmonella* in ready-to-eat foods [24,166,167,168]. Agricultural biocontrol focuses on phytopathogenic bacteria, including *Erwinia amylovora*, *Xanthomonas oryzae*, *P. syringae* and *Ralstonia solanacearum* [149]. Environmental biotechnology applications encompass wastewater treatment, biofilm control and the limitation of antimicrobial resistance gene dissemination [169,170]. Their use in these different sectors also provides useful experience for the further development of phage applications in aquaculture.

## 5. Bacteriophages in Aquaculture

The application of phages as targeted biocontrol agents in aquaculture has expanded over the past four decades, growing from isolated proof-of-concept demonstrations to hundreds of primary studies spanning host species, pathogen targets and production systems. Across both freshwater and marine aquaculture, phages have been characterised and experimentally evaluated against major bacterial pathogens, including *Aeromonas* spp., *Vibrio* spp., *Flavobacterium* spp., *Streptococcus* spp., *Edwardsiella* spp., *Yersinia ruckeri*, *P. plecoglossicida*, *Lactococcus garvieae*, *Photobacterium damselae* and *Hafnia alvei*, with efficacy documented across a wide range of commercially important species [7,34,47,171,172,173,174,175,176,177,178].

Beyond efficacy data, experimental evidence also covers the practical aspects of phage application in aquaculture. Published studies have demonstrated the therapeutic and prophylactic use of phages across freshwater pond culture, recirculating aquaculture systems (RAS), marine cage farming, shrimp hatcheries and larviculture environments [179,180]. Three common administration routes have been investigated: intraperitoneal or intramuscular injection, bath immersion and oral delivery via phage-supplemented or phage-coated feed, each with distinct trade-offs between therapeutic efficacy and operational feasibility at commercial scale [33,47]. Phage stability under the acidic and proteolytic conditions of the fish gastrointestinal tract remains a recognised challenge for oral delivery and is increasingly addressed through biopolymer encapsulation and edible coating systems [7,181]. These observations indicate that successful implementation depends not only on phage efficacy but also on the practicality of delivery methods under commercial farming conditions.

**Figure 2:**
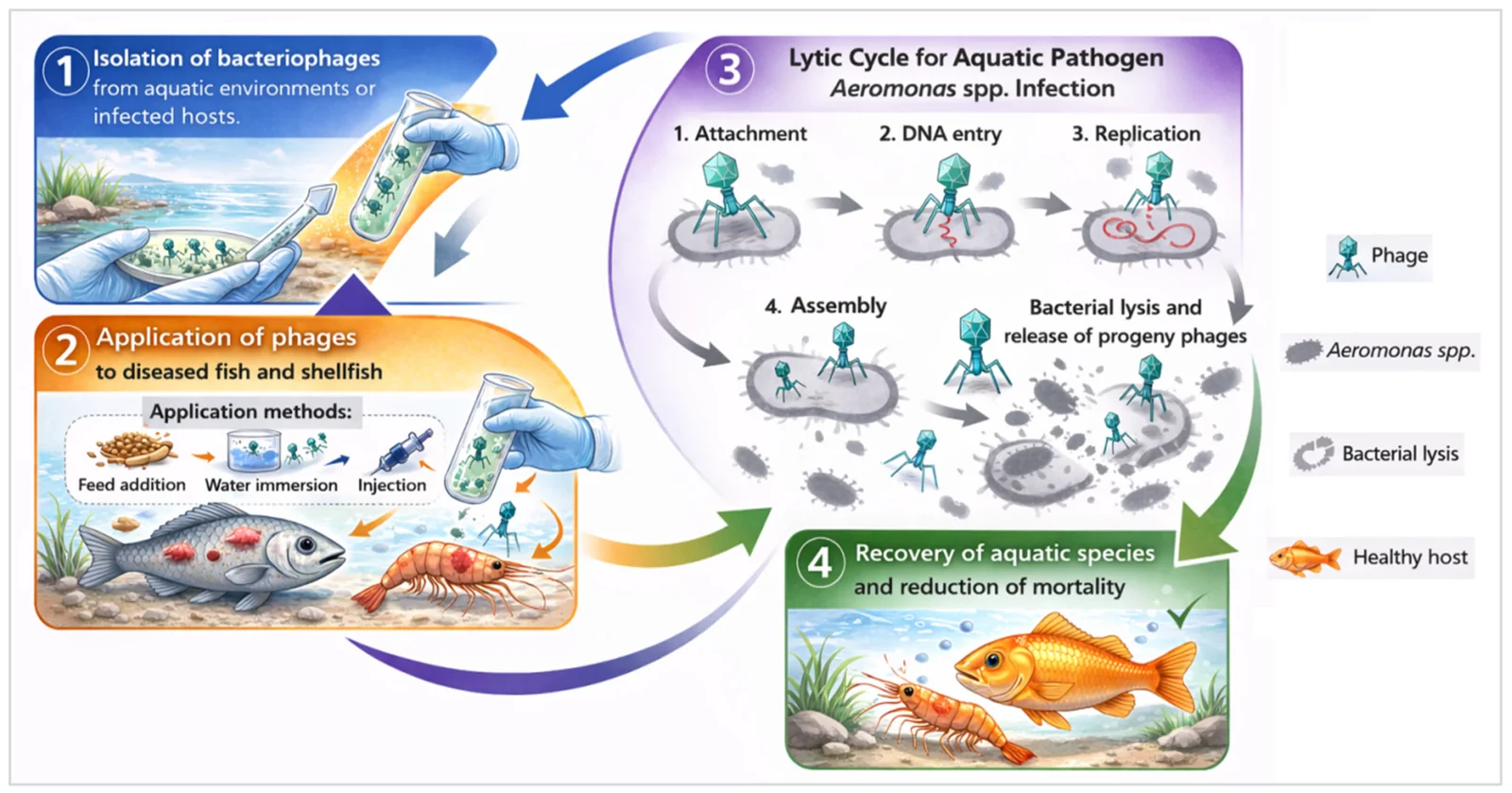
Mechanism of lytic bacteriophage action against *Aeromonas* spp. in aquaculture. The scheme shows the sequence from environmental phage isolation (1) and host-specific adsorption (2), through genome injection, intracellular replication and endolysin-mediated lysis of the bacterial cell (3), to reduction in the bacterial load and recovery of the infected fish (4). This sequence provides the framework against which the studies are organised in Section 6.

Across host species, phages reduced pathogen-induced mortality in aquatic animal models, with prophylactic application often outperforming post-infection therapeutic treatment and phage cocktails reducing the emergence of resistant mutants compared with monophage strategies [34,182,183]. Genome sequencing has become a standard step in phage characterisation, confirming the absence of antimicrobial resistance genes, virulence determinants and lysogenic modules before any candidate phage can advance toward therapeutic or commercial application [145,172,173]. A small but growing number of phage-based products have entered commercial use, although national authorisation for aquaculture remains limited. BAFADOR^®^ (Proteon Pharmaceuticals, Łódź, Poland), a cocktail of seven lytic phages targeting *A. hydrophila* and *P. fluorescens*, is registered as a feed additive in India and available in several European countries, and CUSTUS^®^YRS (ACD Pharma, Leknes, Norway) was approved for aquaculture use in Norway in 2018 to control *Y. ruckeri* in salmonids. Post-harvest and food-safety applications are more widely authorised: ListShield™ (Intralytix, Columbia, MD, USA) received the first phage-based food approval from the United States FDA in 2006, and PhageGuard Listex™ (Micreos, Bilthoven, The Netherlands) holds both FDA/GRAS status and a positive opinion from the European Food Safety Authority. By 2022, around fourteen phage preparations, mostly targeting *Escherichia coli*, *Salmonella* spp. and *L. monocytogenes*, had been approved for food use in the United States, Canada, Israel, Switzerland, Australia, New Zealand and the European Union. Regulatory frameworks remain variable: in Europe, phage products are generally regulated as veterinary medicinal products requiring centralised approval, in the United States the pathway depends on the intended use and authority, and several Asian countries permit more flexible use, which has supported earlier adoption [7,172]. The therapeutic action of a lytic bacteriophage against *Aeromonas* proceeds through a defined sequence of events, summarised in Figure 2. Following isolation from an environment in which its host occurs, typically aquaculture water, sediment or sewage, the phage adsorbs to a specific receptor on the bacterial surface; in *Aeromonas*, lipopolysaccharide and surface-layer proteins act as primary receptors [114]. The phage then injects its genome, redirects host machinery toward viral replication, and phage-encoded endolysins and holins degrade the peptidoglycan cell wall, causing lysis and release of progeny virions [85]. Each cycle both eliminates a bacterial cell and amplifies the phage population, so that at the population level this self-amplifying lysis reduces the bacterial load in the infected host and, in an aquaculture setting, supports recovery of the affected fish.

## 6. Bacteriophages Targeting *Aeromonas* Species

*Aeromonas* spp. cause some of the most persistent bacterial infections in aquaculture and have become a principal target of phage-based disease control [184,185,186,187]. Phage research has concentrated on the two species responsible for most *Aeromonas*-associated losses: *A. salmonicida* [188,189,190,191,192] and *A. hydrophila* [193,194,195], and has since broadened to other pathogenic members of the genus, including *A. dhakensis*, *A. caviae*, *A. jandaei*, *A. schubertii*, *A. bestiarum* and *A. encheleia* [58,196,197,198,199].

**Figure 3:**
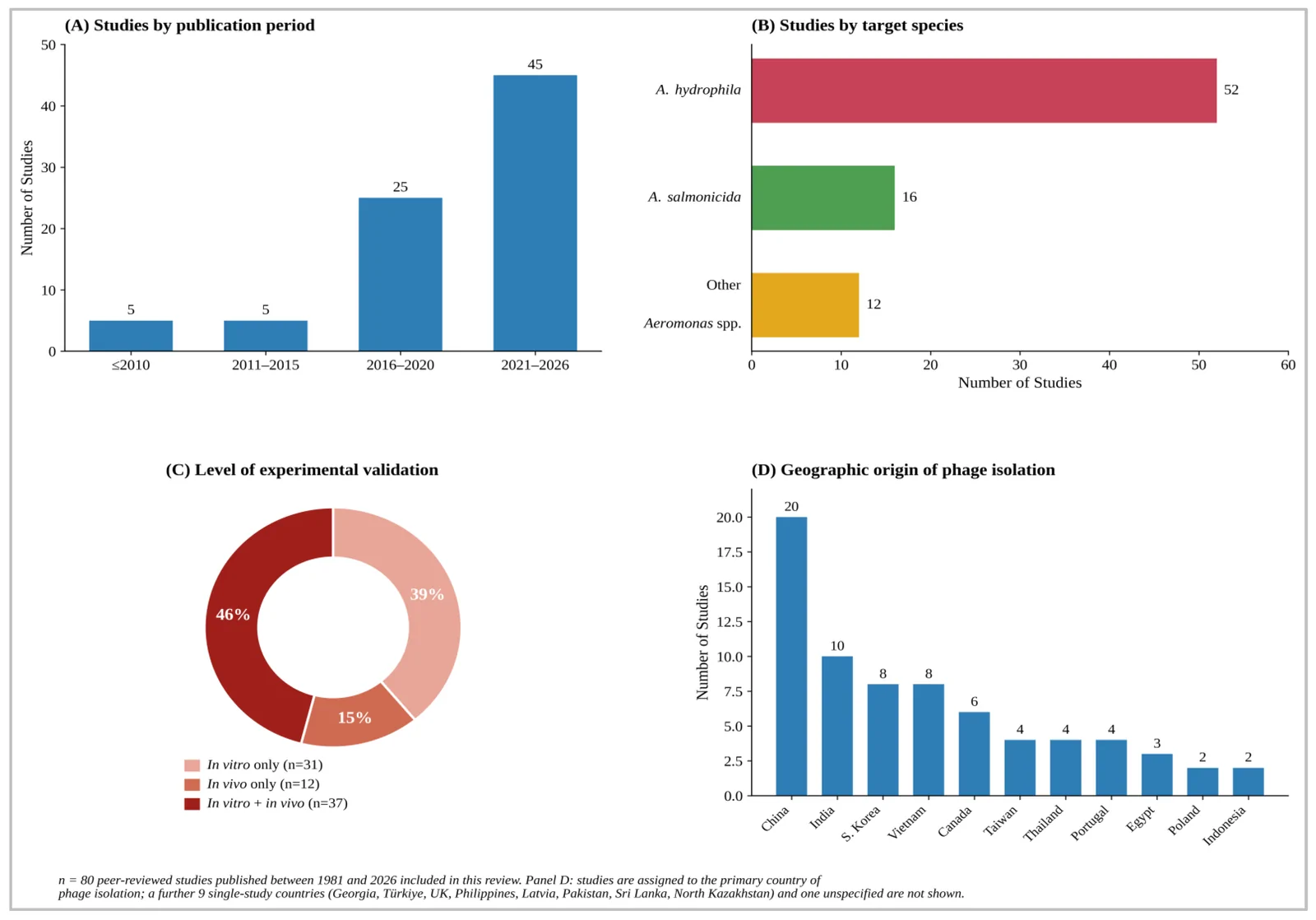
Synthesis of the 80 studies on bacteriophages targeting *Aeromonas* spp. in aquaculture included in this review (1981–2026). (**A**) Number of studies by publication period, showing sustained expansion of the field over four decades. (**B**) Distribution of studies by target species, with *A. hydrophila* representing the most extensively investigated group, followed by *A. salmonicida* and other *Aeromonas* spp. (**C**) Level of experimental validation, classified as in vitro only, in vivo only, or combined in vitro and in vivo assessment. (**D**) Geographic origin of phage isolation, assigned to the primary country reported in each study. A further nine single-study countries and one study with an unspecified country are not shown individually.

The studies also address the stages of the phage life cycle shown in Figure 2 unevenly. Most report in vitro isolation and host range characterisation, with 31 studies limited to this stage. A larger group, 37 studies, combine in vitro work with in vivo evaluation in an aquaculture host, while 12 report in vivo application alone. This distribution shows that phage isolation and host range determination are well represented in the literature, whereas the later stages of delivery formulation and post-treatment monitoring remain comparatively sparse. Four patterns stand out. Research output has grown steadily across four decades, rising from a handful of early reports to 45 studies in the 2021–2026 period (Figure 3), so the field is still expanding rather than slowing. Research effort has been uneven across the genus: *A. hydrophila* accounts for roughly two-thirds of all studies, while *A. salmonicida* and the remaining pathogenic species together make up the rest, in line with their relative weight as aquaculture pathogens. Most studies have moved beyond laboratory characterisation alone, and more than half include in vivo evaluation in aquaculture host species, which points to a translational focus. Phage isolation has clustered in a small number of countries, leaving several large aquaculture-producing regions underrepresented. These patterns set the frame for the species-specific synthesis presented in the following sections.

### 6.1. Bacteriophage Therapy Targeting Aeromonas hydrophila

Research on phages targeting *A. hydrophila* spans more than four decades and represents one of the most extensively studied areas of phage therapy in aquaculture, encompassing studies conducted across different geographic regions and production systems. Rather than examining these studies individually, this section synthesises the evidence published between 1981 and 2026 under recurring themes: host range and specificity, therapeutic and prophylactic efficacy, delivery and formulation strategies, genomic characterisation and safety, and resistance and translational development, including cocktail applications, endolysin-based biocontrol and lysate-derived vaccine platforms. Within each theme, studies are discussed in chronological order to convey how the field has progressed. Advances in whole-genome sequencing and bioinformatic analyses have strengthened the field by enabling routine screening of candidate phages for virulence factors, antibiotic resistance genes and lysogeny-associated elements prior to therapeutic application. Table 1 summarises the principal phages characterised against *Aeromonas* spp., together with their target species, host and source, reported morphotype and taxonomy, application, reported outcomes and references.

*Host range and specificity.* Individual phage isolates targeting *A. hydrophila* generally exhibit a relatively narrow host range and pronounced strain specificity, a characteristic recognised from the earliest studies and consistently reported since. This specificity, while advantageous in sparing non-target bacteria, restricts individual isolates to a limited set of host strains and has made phage cocktails the preferred strategy for achieving broader coverage. The molecular basis of this specificity was clarified through the characterisation of host-recognition determinants in phage Ahp2 [84], and, more broadly, lipopolysaccharide and surface-layer proteins have been identified as the primary receptors mediating adsorption to *Aeromonas* [114]. Against this background of narrow individual ranges, subsequent studies progressively widened coverage through multi-phage and cross-species isolates: Liu et al. [78] reported five phages (N21, W3, G65, Y71 and Y81) active against more than 200 *Aeromonas* isolates and able to prevent and disrupt biofilms, while phage phiA034 was shown to infect fourteen strains across four *Aeromonas* spp. [72,81]. Broad cross-strain activity is therefore achieved through cocktail design rather than through individual isolates, which typically remain restricted in host range.

*Therapeutic and prophylactic efficacy.* Therapeutic efficacy against *A. hydrophila* has been demonstrated consistently across four decades of study, progressing from single-host proof-of-concept experiments to protection in diverse aquaculture species. Across this body of work, the level of protection achieved has depended less on the identity of the individual phage than on a small set of recurring factors: the host species and infection model, the route and dose of delivery, and the timing of administration relative to infection. The earliest evidence came from freshwater fish models, with phage AH1 controlling experimental infection in loach (*Misgurnus anguillicaudatus*) as early as 1981 [55], and treatment later extended to Japanese eel (*Anguilla japonica*) using six lytic phages [56] and to phages pAh1-C and pAh6-C in one of the first large-scale in vivo evaluations, which confirmed protection by both injection and oral administration [57]. From 2010 onwards, a broader panel of phages, including UP87, ΦZH1, ΦZH2, pAh-1, AP1-AP4, Φ2, Φ5, AhSzq-1, AhSzw-1 and AHΦ3, was evaluated across a widening range of host species, including tilapia (*Oreochromis niloticus*), zebrafish (*Danio rerio*), striped catfish (*Pangasianodon hypophthalmus*) and rainbow trout (*Oncorhynchus mykiss*), with most studies reporting reduced disease severity and improved survival [60,61,62,63,64,65,66]. The highest levels of protection have consistently been reported where the delivery route and dose were matched to the host and infection model: complete protection was achieved in rainbow trout with phage MJG by injection or high-dose immersion, accompanied by modulated immune responses and reduced tissue damage [68], while oral feed-based therapy with phage PVN02 reduced mortality from 68.3% to 8.33% in striped catfish [82]. Comparable protection has since been extended to additional hosts and to multidrug-resistant strains, including complete survival in crucian carp (*Carassius auratus*) [92], effective control in whiteleg shrimp (*Litopenaeus vannamei*) with phage AHPMCC7 [96], and improved survival with restored intestinal microbiota homeostasis in grass carp (*Ctenopharyngodon idella*) [107]. Taken together, these studies indicate that protection against *A. hydrophila* is now well established across a broad host range, and that the principal determinants of therapeutic success are the delivery route, the dose and the timing of treatment rather than the particular phage employed.

A recurring and practically important finding is that the timing of administration determines therapeutic success. Across multiple host species, prophylactic or simultaneous administration consistently produced better outcomes than delayed therapeutic intervention [90,91,102]. Prophylactic treatment with phage Ahy-yong1 proved consistently more effective than post-infection therapy [91], and Türe et al. [102] observed the same superiority of prophylactic delivery for an orally delivered four-phage cocktail in rainbow trout. The therapeutic window is not strictly limited to the immediate post-challenge period, however, as phage AhMtk13a retained efficacy when treatment was delayed after infection [90]. Dose is an equally important variable: Kumari et al. [94] showed that a three-phage cocktail (ΦAHBHU12, ΦAHBHU16 and ΦAHBHU19) achieved up to 100% protection through immersion in *Pangasius buchanani*, but that excessive phage doses may trigger the zone phenomenon, underlining the need to optimise dose alongside treatment timing.

*Delivery and formulation.* Delivery strategy is a consistent determinant of therapeutic outcome, and comparative studies have defined the relative merits of the main administration routes. Intraperitoneal injection generally provides the highest level of protection under experimental conditions, whereas oral administration through phage-supplemented feed remains the most practical approach for large-scale aquaculture [99]. This trade-off was quantified directly: Gordola et al. [79] compared routes in Nile tilapia (*Oreochromis niloticus*) and found intraperitoneal injection most effective, followed by oral administration, with bath immersion least effective against systemic infection, while Sankappa et al. [98] reported 100% survival following injection, 95% after immersion and 93% through oral administration using phage AhFM11, indicating that all three routes can be effective when optimised. The practicality of oral delivery was demonstrated by Phumkhachorn and Rattanachaikunsopon [80], who achieved dose-dependent protection through phage-supplemented feed, with the highest dose providing efficacy comparable to oxytetracycline, and by Türe et al. [102], who confirmed translocation of an orally delivered four-phage cocktail beyond the intestinal tract in rainbow trout.

A recognised constraint on oral delivery is phage stability under the acidic and proteolytic conditions of the fish gastrointestinal tract, a limitation identified in early work by Hsu et al. [56] and increasingly addressed through formulation. Robust isolates have been sought to withstand these conditions; for example, phages Ah01 and Ah02 showed high stability across a wide pH range (3–12) and thermal tolerance up to 84 °C [103]. Biopolymer encapsulation and edible coating systems have been applied to protect phages during passage through the gut [181], and Dien et al. [88,89] demonstrated that oxygen nanobubbles enhanced phage retention within host tissues, providing a novel strategy for improving delivery. These developments indicate that successful implementation depends not only on phage efficacy but also on the practicality and stability of delivery under commercial farming conditions.

*Genomic characterisation and safety. *Genome sequencing has been integral to phage development against *A. hydrophila* from early work onward and has become a routine component of candidate evaluation. Wang et al. [59] provided one of the first examples, sequencing pAh6-C and confirming the absence of lysogeny-associated and virulence genes, thereby establishing genome-informed candidate selection for therapeutic use. As sequencing became standard, the same screening was applied to successive isolates: Cao et al. [67] confirmed the absence of antibiotic resistance, virulence and lysogeny-associated genes in phage MJG, and comparable screening of phages such as Akh-2 and vB_AhM_PVN02 consistently confirmed the absence of these elements [74,75]. This screening supported the broader development of phage cocktails, endolysins and other phage-derived antimicrobial approaches [63,64,65,66]. Genome analysis also began to inform candidate selection prospectively, with Kazimierczak et al. [69] demonstrating computational prediction of phage lifestyle as a means of identifying strictly lytic candidates, and subsequent studies consistently confirming the absence of virulence, antibiotic resistance and lysogeny-associated genes in candidate therapeutic phages [97,99,101].

Genomic studies have also progressively expanded the known diversity and taxonomic placement of these phages, with contributions from India and Vietnam characterising phages AhFM4, AhFM5, AhyVDH1 and the PVN03-PVN05 group and highlighting biofilm-disruption capacity and regional genomic diversification [76,81,87]. Several isolates introduced novel taxa: Ye et al. [95] described phiA034 as the first *Aeromonas* phage assigned to the family *Casjensviridae* and the jumbo phage vB-AhyM-AP1 was described among the isolates that expanded the known diversity of these phages [86]. Alongside this expansion, investigations of temperate and prophage-derived lineages by Wang et al. [170] indicated the evolutionary complexity of these phages and reinforced the importance of thorough genomic screening before therapeutic use, as illustrated by prophage-derived lineages such as phiA051 [170]. Biosafety was further supported at the functional level: Feng et al. [93] showed that phage PZL-Ah152 reduced bacterial burden without disrupting intestinal microbiota.

*Resistance and translational development.* The rapid emergence of phage-resistant mutants is a recurring limitation, recognised early and repeatedly confirmed. Phage-resistant *A. hydrophila* mutants have been observed to display reduced virulence [57], while the rapid emergence of resistant mutants in vitro has been documented repeatedly [71,72], reinforcing the need for cocktail strategies to overcome resistant bacterial strains [76,81,87]. Phage cocktails have consequently become the principal means of limiting resistance, with Yu et al. [92] showing that a two-phage cocktail (vB_AhaP_PZL-Ah8 and vB_AhaP_PZL-Ah1) reduced resistant-mutant emergence while removing established biofilms, and a 7.7 log CFU/mL reduction was achieved in contaminated cockles, with cocktails generating lower resistance frequencies than individual phages [83]. A complementary approach has been the combination of phages with antibiotics: synergistic activity has been reported between phage AHP-1 and chloramphenicol [77], while Zhang et al. [106] demonstrated synergy between jumbo phage Z90 and ampicillin, providing evidence that phage-antibiotic combinations may reduce antibiotic requirements while maintaining efficacy against multidrug-resistant *A. hydrophila*.

The therapeutic repertoire has also expanded beyond intact virions towards phage-derived products. The broad-spectrum endolysin PlyD4 provided 75% survival in a zebrafish model and represented the first prophage-derived endolysin reported against *A. hydrophila* [85]; subsequently, the first recombinant endolysin with permeabiliser-independent activity against this bacterium was reported, and the Hol46-Lys17 holin-endolysin system was described as a potent agent against resistant *A. hydrophila*, clearing infection and improving carp survival [104]. Phage lysate-based vaccines have become a further platform. Liang et al. [100] demonstrated that a lysate vaccine derived from phage PZY-Ah achieved 88.89% protection in crucian carp and outperformed conventional formalin-inactivated vaccines, and together with earlier observations of phage-derived lysates [73], these findings indicate that such lysates may offer an alternative platform for inactivated vaccine development. At the translational level, Schulz et al. [70] evaluated a commercial phage cocktail in rainbow trout and reported both therapeutic and prophylactic benefits, while studies from Indonesia supported locally adapted farm-derived phages as practical biocontrol agents under aquaculture conditions [105]. Taken together, phage cocktails, prophylactic timing and oral feed-based delivery represent the most translationally relevant strategies, while endolysins and phage lysate vaccines extend the repertoire beyond intact virions; the principal remaining challenges are the narrow host range of individual isolates, the rapid emergence of resistant mutants and the limited number of large-scale field validations under commercial conditions.

### 6.2. Bacteriophage Therapy Targeting Aeromonas salmonicida

Phage research targeting *A. salmonicida*, the causative agent of furunculosis, spans more than four decades and has progressed from studies of phage-host interactions to optimised phage cocktails and in vivo therapeutic applications. The principal phages characterised against this species are summarised in Table 1, and the evidence is organised under the same recurring themes and chronological approach applied above.

*Host range and specificity.* Bacterial surface architecture has been recognised as the principal determinant of host specificity in *A. salmonicida* from the earliest studies. Rodgers et al. [108] developed the first phage typing framework for this species and demonstrated that lipopolysaccharide (LPS) composition and colony morphotype strongly influenced phage susceptibility, a conclusion later confirmed through molecular and genomic studies. The molecular basis was subsequently clarified by work showing that mutations affecting LPS biosynthesis were strongly associated with phage resistance and that the A-layer surface protein can mask receptor sites and reduce adsorption efficiency, establishing LPS and the A-layer as key determinants of susceptibility [114]. The same work demonstrated substantial genomic diversity among *A. salmonicida* phages and showed that host range could not be reliably predicted from geographic co-isolation with target strains, indicating the need for empirical phage selection during cocktail design. Host range varies widely among isolates: T7-Ah (HER212) was described as the first podovirus reported to infect both psychrophilic and mesophilic *A. salmonicida* strains, indicating value for broad-spectrum formulations [119], whereas JELG-KS1, a genetically distinct podophage from wastewater, exhibited a highly restricted host range [123].

*Therapeutic and prophylactic efficacy*. Therapeutic and prophylactic efficacy has been demonstrated across a range of salmonid and marine hosts, though outcomes have varied with the phage-host combination, the delivery route and the timing of treatment. The first successful demonstration of phage-based prophylaxis in a salmonid model (*Salvelinus fontinalis*) was reported by Imbeault et al. [109], who showed that phage HER110 substantially reduced mortality and delayed disease progression. Efficacy was not universal, however: found that a three-phage cocktail (O, R and B) failed to protect Atlantic salmon (*Salmo salar*) despite successful delivery and the absence of adverse effects, highlighting the importance of phage-host compatibility and treatment timing [110]. Consistent protection has nonetheless been reported in commercially important species: Silva et al. [113] demonstrated that phage AS-A provided complete protection against *A. salmonicida* in juvenile Senegalese sole (*Solea senegalensis*), representing one of the earliest successful applications in a non-salmonid marine species. Efficacy against resistant bacterial populations has also been confirmed, with a lytic phage showing strong activity against multidrug-resistant *A. salmonicida* in both in vitro and in vivo models [118], and phage vB_AsM_ZHF reducing bacterial burden, improving survival and attenuating inflammatory responses in turbot (*Scophthalmus maximus*) infected with *A. salmonicida* subsp. *masoucida*, indicating benefits extending beyond direct bacterial killing [121].

*Delivery and formulation.* Studies addressing delivery and environmental behaviour have focused on the stability and ecological safety of phage preparations under aquaculture conditions. Pereira et al. [111] demonstrated that phage AS-1 remained stable under marine aquaculture conditions and produced no detectable disruption of the dominant bacterial community structure, providing early evidence that these viruses could be deployed without major impacts on non-target microbial communities. Environmental studies have since confirmed acceptable persistence and minimal disruption of non-target communities, supporting the safe deployment of well-characterised preparations in both closed and semi-open aquaculture systems [111,115]. Across these studies, successful outcomes were most consistently achieved when phages were selected according to local bacterial populations and applied before widespread systemic infection had developed.

*Genomic characterisation and safety.* Genomic characterisation has defined and progressively expanded the known taxonomic diversity of *A. salmonicida* phages and progressively expanded their known diversity. Early genomic work established the predominance of the myovirus lineage: characterisation of phiAS4 revealed a T4-like myovirus genome typical of large *Aeromonas*-infecting phages, and Kim et al. [112] described PAS-1 as a lytic myovirus active against multiple *A. salmonicida* subspecies, including antibiotic-resistant isolates. Subsequent studies expanded diversity beyond this repertoire: Yang et al. [117] described AsXd-1 as the first representative of the genus *Hk97virus* reported for *A. salmonicida*, demonstrating that environments such as food-processing wastewater may serve as reservoirs of novel phage diversity, while the first podoviruses were added to the characterised repertoire through T7-Ah (HER212) [119] and the wastewater isolate JELG-KS1 [123]. Genomic screening has also supported safety and rational selection, exemplified by phage MQM1 (vB_AsaP_MQM1), which showed preferential activity against Prophage 3-carrying strains while lacking integrase, virulence and antibiotic resistance genes [122]. An additional immunological consideration was the detection of neutralising antibodies following PAS-1 administration, suggesting that host immune responses may influence phage persistence during prolonged treatment [112].

*Resistance and translational development.* Resistance management has been the principal driver of the shift from single-phage treatments towards rationally designed cocktails. Early support for this approach came from the observation that phage-resistant mutants frequently exhibited reduced fitness and remained susceptible to alternative phages [109]. Individual phages were subsequently shown to differ substantially in burst size, latent period and resistance-suppression capacity, indicating complementary functions within cocktails [115], and Chen et al. [116] demonstrated that multi-phage combinations consistently outperformed individual phages in bacterial suppression and treatment durability. Cocktail design became increasingly genome-informed: Hosseini et al. [120] evaluated multiple combinations against genetically diverse *A. salmonicida* subsp. *salmonicida* isolates and identified a four-phage formulation (Cocktail T) providing the most durable suppression, but also revealed that strains carrying Prophage 3 exhibited reduced sensitivity through superinfection exclusion. This limitation was directly addressed by phage MQM1, which showed preferential activity against Prophage 3-carrying strains [122], providing a clear example of how genomic information from both viral and bacterial genomes can be incorporated into rational cocktail design.

Taken together, the evidence indicates several recurring themes for practical application. Myoviruses dominate the characterised repertoire and account for most successful in vivo applications, although recently characterised podoviruses, including T7-Ah and MQM1, have expanded the diversity available for cocktail design [119,122]. Multi-phage cocktails consistently outperform single-phage treatments in suppression, resistance management and durability [115,116,120], and the geographic and genetic relationship between candidate isolates and target populations is an important determinant of efficacy, as shown by the contribution of the French-isolated phage HER110 to cocktail performance against Quebec strains [120]. The prophage content of target populations, particularly the prevalence of Prophage 3 among North American isolates, represents a confirmed obstacle through superinfection exclusion [120,122], indicating that genomic surveillance of target populations should inform phage selection. The evidence supports rationally designed, genomically screened and regionally validated phage cocktails as a promising non-antibiotic approach for furunculosis management, while prophage-mediated resistance and large-scale field validation remain important priorities for future research.

### 6.3. Bacteriophage Therapy Targeting Other Aeromonas Species

Bacteriophage research targeting *Aeromonas* spp. other than *A. hydrophila* and *A. salmonicida* has expanded during the past decade, although the available literature remains comparatively limited. The evidence is synthesised here under the same themes used in the preceding sections, with the understanding that the smaller evidence base for these species places most of the available data within the host range, efficacy and resistance themes.

Host range and specificity. Early studies of phages targeting these species revealed a recurring trade-off between strong lytic activity and narrow host specificity, a pattern observed in phages infecting both *A. punctata* and *A. veronii*, where efficient bacterial lysis was frequently accompanied by restricted strain coverage [124,125]. Subsequent research focused on overcoming this limitation through broader-host-range isolates: the lytic phage phiA034 infected 14 strains across multiple species, extending beyond the narrow host profiles reported for earlier phages, while genomic analysis confirmed the absence of virulence and antibiotic resistance genes [95]. The continued discovery of novel lineages, including the identification of AerV_348 as a previously unclassified lineage within aquatic *Caudoviricetes*, indicates that the diversity of environmental phages remains incompletely explored and may provide additional therapeutic candidates with expanded host ranges [135].

Therapeutic and prophylactic efficacy. Therapeutic efficacy has been demonstrated across several of these species, with cocktail formulations consistently outperforming single-phage applications in vivo. A combination of phages pAEv1810, pAEv1812 and pAEv1818 achieved survival rates approaching 80% in gibel carp (*Carassius auratus gibelio*) while preserving gut microbiota composition and physiological function [126], and additional *A. veronii* phages, including pAEv1 and AVP-type phages, reduced bacterial burden, improved survival, and demonstrated favourable environmental stability and biofilm-control properties [127,128,129,130]. Efficacy has also been reported in further species: phages targeting *A. schubertii* improved host survival and exhibited strong environmental resilience [129], and cross-system validation using phage vB_AceP_PAc demonstrated reduced inflammation and improved gut barrier function in a mammalian model, suggesting that some therapeutic principles may extend beyond aquaculture [134].

Delivery and formulation. Delivery-relevant properties, particularly environmental stability, have been reported for several isolates. Novel phages such as vB_AerC_LA93P1 and vB_AerC_LA93P2 demonstrated high burst sizes and strong stability under aquaculture-relevant conditions [132], and the favourable environmental stability of pAEv1 and AVP-type phages has similarly been noted as supporting their practical deployment [127,128,129,130].

Genomic characterisation and safety. Genomic screening has accompanied the characterisation of candidate phages for these species, confirming the absence of virulence and antibiotic resistance genes in isolates such as phiA034 [95]. The identification of AerV_348 as a previously unclassified lineage within aquatic Caudoviricetes further indicates that the genomic diversity of environmental *Aeromonas* phages remains incompletely characterised [135].

Resistance and translational development. As in the other species groups, narrow host range and resistance development remain the principal limitations, and cocktail and combination strategies are the main responses. Partial bacterial regrowth under prolonged phage exposure has highlighted the continuing challenge of resistance development [133]. Phage-antibiotic combinations appear particularly valuable against multidrug-resistant isolates: in *A. dhakensis*, individual phages reduced bacterial counts by up to 6.67 log CFU/mL, but complete bacterial elimination was achieved only when a two-phage cocktail was combined with amoxicillin at a sub-inhibitory concentration (32 µg/mL, one-half the MIC), providing evidence that phage-antibiotic synergy may outperform monophage therapy against multidrug-resistant infections [131]. Across these species, the narrow host range of individual isolates, while cocktails generally provide broader coverage and more sustained bacterial control. The diversity of environmental phages is still incompletely represented in current collections. Larger phage collections and testing under commercial aquaculture conditions are therefore still needed.

## 7. Advantages, Limitations and Future Perspectives

Although the studies reviewed differed substantially in host species, bacterial strains and production systems, a number of recurring patterns appeared across the species. Research on *A. hydrophila*, *A. salmonicida* and other *Aeromonas* spp. followed a similar progression from phage isolation and characterisation towards genomics-guided candidate selection, optimised delivery approaches and increasingly sophisticated therapeutic applications. The studies now point more towards improving phage selection, formulation and delivery than simply identifying additional candidates.

The evidence reviewed in this study demonstrates several advantages of bacteriophage therapy over conventional antibiotic-based approaches for the management of *Aeromonas* infections in aquaculture. The most important of these is host specificity, which allows the selective elimination of target pathogens while preserving the resident microbiota. However, this same specificity is a double-edged property: because individual phages infect only a narrow range of strains, their broad application across diverse pathogen populations is inherently limited. Unlike broad-spectrum antibiotics, which may disrupt microbial communities and contribute to dysbiosis, phages generally maintain microbial homeostasis while reducing pathogen abundance [12,200]. This characteristic is particularly relevant in aquaculture systems where microbiome stability influences animal health, growth performance, and disease resistance. In addition, phages replicate at the site of infection in response to bacterial density, creating a self-amplifying therapeutic effect that differs fundamentally from conventional antimicrobials [201,202,203,204]. A meta-analysis of aquatic animal studies further demonstrated that phage therapy can significantly reduce mortality and provide efficacy comparable to antibiotic treatment under experimental conditions [182].

Beyond their antibacterial activity, phages offer several practical and ecological advantages. Numerous studies reviewed here showed that phage cocktails improve bacterial coverage, reduce resistance emergence, and provide more durable therapeutic outcomes than single-phage preparations [92,116,120]. Phage-encoded depolymerases and related enzymes can disrupt bacterial biofilms, thereby improving access to bacterial cells that are often protected from antibiotic exposure [201]. Phage-antibiotic synergy has also proved a promising strategy, particularly against multidrug-resistant *Aeromonas* strains, where combined treatments frequently outperform either approach alone [131,205]. Unlike antibiotics, these viruses do not leave toxic residues in aquatic environments or edible tissues, and their natural occurrence in aquatic ecosystems facilitates the identification of new candidates when resistance emerges. These characteristics support the growing role of bacteriophages as environmentally compatible disease-management tools within sustainable aquaculture and One Health frameworks [205].

At the same time, several biological and practical limitations continue to restrict large-scale implementation. The narrow host range of many phages remains the most important biological constraint, as individual isolates often infect only a subset of strains within a target species [202,203,204]. Although phage cocktails consistently improve bacterial coverage and reduce resistance emergence, effective formulations frequently require prior characterisation of local pathogen populations [92,116,120]. Bacterial resistance can arise through receptor modification, restriction-modification systems and CRISPR-Cas-mediated defence mechanisms, while environmental factors such as temperature, salinity, ultraviolet radiation and gastrointestinal conditions may reduce phage stability and efficacy [12,206]. In addition, most published studies have been conducted under laboratory or experimental challenge conditions, whereas large-scale field evaluations under commercial farming conditions remain comparatively limited [44,182].

Several findings from this review also highlight important translational limitations. Standardised pharmacokinetic and pharmacodynamic frameworks for phages in aquatic animals remain poorly developed, complicating dosage optimisation and treatment standardisation [180]. Regulatory pathways likewise remain fragmented across different regions, despite recent progress in Europe, North America and Asia toward the development of dedicated guidance documents and approval frameworks [168,204,207,208,209,210]. The economic feasibility of large-scale phage production, formulation and distribution also requires further attention, particularly in regions where *Aeromonas*-associated disease burdens are highest and financial resources are limited [82,177].

Future progress will likely depend less on the discovery of additional phages and more on improving the selection, formulation and deployment of existing candidates. One of the most consistent conclusions emerging from studies of *A. hydrophila*, *A. salmonicida* and other spp. within the genus is that phage cocktails provide broader bacterial coverage, lower rates of resistance emergence and more durable therapeutic outcomes than single-phage preparations [92,116,120]. Increasingly, genomic screening of both viral and bacterial hosts is being incorporated into cocktail design, particularly following evidence that bacterial prophages, host-range variation and local strain diversity can substantially influence treatment success [114,120,122]. Future cocktail design is therefore likely to rely increasingly on genomic information from both the phage and the target bacterial population. Advances in delivery technologies represent another important area of development. Oral administration through medicated feed has repeatedly demonstrated practical value under aquaculture conditions [57,82,102], while encapsulation systems and protective formulations may improve phage stability during storage and gastrointestinal transit [208]. At the same time, therapeutic options are expanding beyond intact viral particles. Phage-derived endolysins have shown promising activity against this genus, including multidrug-resistant strains [85,201], whereas phage lysate vaccines have demonstrated encouraging protective efficacy and immunostimulatory properties in experimental fish models [73,100]. These developments suggest that future disease-management strategies may increasingly combine phages with complementary phage-derived antimicrobial products.

Commercial applications are beginning to appear. Products such as BAFADOR^®^ and aquaPHIX™ demonstrate that phage-based disease control can be incorporated into commercial aquaculture systems through feed-delivery technologies and multi-phage formulations [179,206]. Continued progress in genomics, metagenomics, computational biology and synthetic biology is expected to further support these developments by improving phage selection, expanding host-range coverage and facilitating the development of next-generation therapeutic platforms [171,183,211,212]. These advances indicate that bacteriophage therapy is gradually transitioning from an experimental approach toward an integrated component of sustainable aquaculture health management.

Several further limitations should be considered when interpreting this review. Screening and study selection were performed by a single reviewer, and selection decisions were not independently verified, which introduces a risk of selection bias. The review was not registered in a protocol database, and no formal risk-of-bias appraisal of the included studies was performed, since the work was conducted as an integrative rather than a systematic review. The search was restricted to English-language publications indexed in three databases, so relevant studies published in other languages, or in sources not indexed by PubMed, Scopus or Web of Science, may have been missed.

## 8. Conclusions

Over the past four decades, phage therapy against *Aeromonas* infections in aquaculture has progressed from experimental proof-of-concept studies to a diverse therapeutic platform. The evidence reviewed between 1981 and 2026 shows that phages can reduce disease burden across multiple species and aquaculture hosts, offering a realistic alternative to conventional antibiotic-based management. Across studies targeting *A. hydrophila*, *A. salmonicida* and other *Aeromonas* spp., the same priorities recur: cocktail formulations over single phages, prophylactic over delayed application, genomic screening as a routine safeguard, and a therapeutic repertoire now extending to endolysins, phage-antibiotic combinations and lysate-based vaccines.

The field is no longer constrained by a shortage of candidate phages but by the steps that turn them into reliable products: regionally curated collections, standardised efficacy and safety frameworks, optimised delivery and the integration of genomic surveillance into therapeutic design. Resolving the remaining host-range, resistance, pharmacokinetic and regulatory gaps will determine how far phage-based control can move from controlled trials into commercial practice. On current evidence, phage therapy shows clear potential as a non-antibiotic strategy for sustainable aquaculture health management, with a direct contribution to reduced antibiotic use, improved aquatic animal health and the wider goals of the One Health framework.

## Figures and Tables

**Table 1:** Summary of bacteriophages targeting *Aeromonas* spp.: morphological, genomic and in vivo/in vitro therapeutic properties (1981–2026).

Phage Name	Target *Aeromonas* spp.	Host/Source	Country	Reported Morphotype/Taxonomy	Application	Main Outcome	References
***Aeromonas hydrophila***							
AH1	*A. hydrophila*	*Misgurnus* *anguillicaudatus*	Taiwan	Myovirus	*in vivo*	First isolation of AH1. Complete elimination of pathogenicity after phage pretreatment; mortality reduced from 65% in untreated controls to 0% in AH1-treated loach.	[55]
A1, A2	*A. hydrophila*	*Anguilla japonica*	Taiwan	*Caudoviricetes*	*in vitro*	Phage represents viable, antibiotic-free strategy for managing bacterial infection in eels. Parameters such as multiplicity of infection (MOI) and temperature were found to exert strong influence on performance. Phages decreased after 2 h. In pond water, phage treatment reduced 250-fold *A. hydrophila* population in 8 h.	[56]
pAh1-C pAh6-C	*A. hydrophila*	*M. anguillicaudatus*	South Korea	Myovirus	*in vivo*	Efficient lytic activity against *A. hydrophila* loach, reduced mortality in challenge trials. Administered by immersion and injection. Useful in phage cocktails; latent periods of phages were estimated to be approximately 30 min (pAh1-C), 20 min (pAh6-C).	[57]
pAh6-C	*A. hydrophila*	*M. anguillicaudatus*	South Korea	Myovirus	*in vivo*	pAh6-C represents genetically stable, virulent phage with strong potential as biocontrol agent against antibiotic-resistant *A. hydrophila* in fish farming.	[57,58]
UP87	*A. hydrophila*	*Oreochromis* *niloticus*	Philippines	Myovirus	*in vivo*	There were no significant differences between group treated with oxytetracycline and group treated with UP87. Phage application led to a reduction of 100% mortality at 72 h of trial.	[59,60]
ΦZH1, ΦZH2	*A. hydrophila*	*O. niloticus*	Egypt	Podovirus	*in vitro/in vivo*	Phages ΦZH1 and ΦZH2 administered via injection 24 h prior were found marked reduction to 18% in treating fish infected with *A. hydrophila*, fish mortality was monitored over 15-day observation time. In vivo findings showed untreated infected fish exhibited 68% mortality.	[61]
pAh-1	*A. hydrophila* KCTC 12487	*Danio rerio*	South Korea	Myovirus	*in vivo*	Administered by immersion and injection. During 48 h incubation period, zebrafish infected with *A. hydrophila* showed 96.67% mortality rate, while fish treated with pAh-1 exhibited lower mortality rate of 56.67%, achieving 43.33% survival rate.	[62]
AP1, AP2, AP3, AP4	*A. hydrophila*	*O. niloticus*	Alexandria, Egypt	not provided	*in vitro/in vivo*	Histopathology revealed that liver and gill tissues of phage-treated fish showed partial recovery compared to severe degeneration, hepatocyte disruption, and lamellar separation observed in untreated infected fish, supporting therapeutic promise of marine phage AP2 for controlling MAS in tilapia. Results: 94% elimination of *A. hydrophila* compared to phage infectivity under basal conditions.	[63]
ΦF2, ΦF5	*A. hydrophila*	*Pangasianodon hypophthalmus*	Vietnam	Myovirus	*in vitro/in vivo*	Phage treatments applied to bacterial strains during infestation of catfish resulted in survival rates of tested fishes with up to 100% compared (8-day) to 18.3% survival observed in control experiments.	[64]
AhSzq-1, AhSzw-1	*A. hydrophila* KT998822	Seawater	China	*Siphoviridae*/*T5virus*	*in vitro*	Two phages specific to *A. hydrophila*; 88% DNA and 83% protein sequence similarity to each other; virion morphology, genome size and terminal repeats consistent with T5-like phages; phylogenetic and proteomic analyses placed both within the *T5virus* genus	[65]
AHΦ3	*A. hydrophila*	*Oncorhynchus mykiss*	Iran	not provided	*in vivo*	AHΦ3 showed activity against its specific host *A. hydrophila*; phage treatment was reported to improve the survival rate of challenged rainbow trout, indicating phages as a possible control option for aeromoniasis	[66]
MJG	*A. hydrophila*	*O. mykiss*	Harbin, China	Podovirus (*Sp6virus*)	*in vitro/in vivo*	MJG had activity at temperatures 10–60 °C, pH 2 to10, and its latent and rise periods were 30–40 min. MJG treatment restored liver tissue damages, abolishing clinical signs of infection. Intraperitoneal injection or high-dose immersion fully prevented mortality, and high-dose oral delivery achieved roughly 70% survival.	[67,68]
25AhydR2PP 50AhydR13PP 60AhydR15PP	*A. hydrophila, A. salmonicida, A. sobria*	*O. mykiss,* Envronmental isolates	Poland	Podovirus, Myovirus	*in vitro*	Complete genome sequences of *Aeromonas*, *Pseudomonas* phages development in aquaculture. Several isolated phages exhibited relatively narrow but complementary host ranges, lysing between 8% and 51% of tested field isolates; six phages fulfilled key safety and lytic criteria for potential therapeutic application, theoretically covering approximately 41% of *Aeromonas* spp.	[69]
50AhydR13PP 60AhydR15PP 25AhydR2PP	*A. hydrophila*	*O. mykiss*	Poland	not provided	*in vivo*	Both prophylactic and therapeutic tests lasted 14 days with bacteriophage cocktail. 24 h after infection, intraperitoneal injection and immersion application reduced mortality by approximately 25% compared to infected controls. 36% higher survival rate was achieved compared to untreated infected fish.	[70]
CT45P, TG25P	*A. hydrophila*	*P. hypophthalmus*	Vietnam	not provided	*in vitro*	Phage TG25P effectively inactivated *A. hydrophila* (decreased appr. 3 log CFU/mL after 8 h). Phage CT45P was revealed to be more pH-sensitive than phage TG25P at pH 4–5.	[71]
TG25P, CT45P, TG22P, TG23P	*A. hydrophila* A1	*P. hypophthalmus*	Vietnam	not provided	*in vitro*	Cocktail TG25P, CT45P was subjected to inactivate *A. hydrophila*. Phage-resistant bacteria emerged 8 h post-treatment. More phage is not automatically superior if resistance mechanisms are heterogeneous.	[72]
pAh6-C	*A. hydrophila* JUNAH	*Cyprinus carpio*	South Korea	Myovirus	*in vivo*	Phage lysate vaccine elicited stronger immune responses and higher survival in common carp than a formalin-killed cell vaccine; both antigens were delivered by PLGA encapsulation, and high-dose lysate formulations produced the highest agglutination titres.	[73]
Akh-2	*A. hydrophila* KCTC 2358	*M. anguillicaudatus*	Geoje Island, South Korea	Siphovirus	*in vitro/in vivo*	Fish immersion-treated phage therapy showed improved survival with a cumulative mortality rate stabilising around 56–57% over 96 h, which means appr. 43% relative protection. The surviving fish in the treated group showed reduced clinical signs.	[74]
vB_AhM_PVN02	*A. hydrophila* 4.3T, 4.4T	*P. hypophthalmus*	Vietnam	Myovirus	*in vitro*	First broad genomic characterisation of *A. hydrophila* phages isolated from striped catfish farms. PVN02 phage with strong lytic capacity, short latent, moderate size, and high infectivity controls MAS.	[75,76]
AHP-1	*A. hydrophila*	*Carassius carassius*	South Korea	Myovirus	*in vitro*	Phage adsorption showed that 81% of phage particles were adsorbed onto *A. hydrophila* in the first 25 min. Bacterial inactivation was time- and MOI-dependent. At an MOI of 10, the phage showed higher growth inhibition against *A. hydrophila* when compared with MOIs of 0.01, 0.1 and 1.	[77]
N21, W3, G65,Y71,Y81	*A. hydrophila*	Fish ponds and polluted rivers	Nanjing	Myovirus	*in vitro*	All phages reduced *A. hydrophila* growth at an MOI of 0.01, 0.1, 1 and 10. Bacterial regrowth 12 h after incubation with N21, W3, and Y71. G65 and Y81 showed bacterial killing effect and prevented *A. hydrophila* biofilm; G65, W3, and N21 were able to scavenge biofilms effectively.	[78]
VB_AehMDM8VB_AehMDM12 VB_AehMDM14VB_AehPDM11	*A. hydrophila*	Water, *O. niloticus*	not provided	Myovirus, Podovirus	Not stated	All exhibited reduction symptoms, significant (*p* < 0.05) decrease in bacteria and increase in phage. IP route showed marked activity in reducing bacterial load. Efficacy of oral route depends on the fish’s intake of feeds, whereas bath route was least effective.	[79]
PAh4	*A. hydrophila* UR1, UR10, ATCC 700183	*O. niloticus*	Thailand	Myovirus	*in vitro/in vivo*	Highest bacteriophage dose (10^8^ PF/g) in feed, orally, markedly reduced cumulative mortality and showed protective efficacy comparable to oxytetracycline treatment. No adverse effects were observed in fish fed with bacteriophage-supplemented diets.	[80]
AhyVDH1	*A. hydrophila* 4572	not provided	China	Myovirus	*in vitro,* genomic characterisation	*A. hydrophila* growth started approximately 2.5, 2, 1 h post-infection, depending on MOI; marked inhibition lasted up to 12 h. Probably due to the appearance of phage-resistant mutants, growth was observed appr. 12 h later. AhyVDH1 infected only one of the seven tested *Aeromonas* strains, indicating a narrow host specificity.	[81]
PVN02	*A. hydrophila* 4.4T	*P. hypophthalmus*	Vietnam	not provided	*in vivo/in vitro*	Without the existence of the phage, the highest mortality rate was 68.3 at highest density of bacterial suspension, and mortality rate at highest density of bacterial suspension was reduced to 8.33% or 16.67% at phage dose of log 6.2 or log 4.2 PFU/g.	[82]
AH-1, AH-4, AH-5	*A. hydrophila* ATCC7966	*Cerastoderma edule*	Portugal	Myovirus	*in vitro/in vivo*	Phages AH-4 and AH-5 exhibit strong lytic activity against *A. hydrophila*, particularly under controlled in vitro conditions, but phage AH-1 has max reduction of 7.7 log colony-forming units CFU/mL.	[83]
pAh-1, PlyD4	*A. hydrophila*	Isolated from sewage water	Taiwan, China	Myovirus	*in vitro/in vivo*	Phage adsorption showed that 96% of phage adsorbed to *A. hydrophila* in 18 min. Ahp2 lysed *A. hydrophila* in 3.5 h at an MOI of 0.0001 does not lysogenize its host. Phage-derived endolysins such as PlyD4 represent promising alternatives to antibiotics for controlling bacteria.	[84,85]
vB-AhyM-AP1	*A. hydrophila*	Raw sewage	India	not provided	*in vivo*	Phage added directly to rearing water. These phages lacked virulence and antibiotic resistance genes, but they did have lysis capability against *A. hydrophila* and associated biofilms.	[86]
AhFM4, AhFM5	*A. hydrophila*	*D. rerio*	India	Myovirus	*in vitro*	AhFM4 showed adsorption efficacy of 96% in 30 min to host, and no phages were detected in supernatant at 40 min. AhFM5 showed low adsorption efficacy, 70%, in 30 min to the host cells, and no phages were detected in the supernatant at 55 min.	[87]
pAh6.2TG	*A. hydrophila*	*O. niloticus*	Thailand	*Chaseviridae*	*in vivo*	pAh6.2TG eliminated 83% of tested *A. hydrophila* strains and increased survival rate of tilapia under infection stress by up to 80%. Administered by immersion, oral route and bath. It infected relatively stably at pH 7–9, temperature 0–40 °C and salinity 0–40 ppt.	[88]
pAh6.2TG	*A. hydrophila* BT14	*O. niloticus*	Thailand	*Chaseviridae*	*in vivo*	Ozone nanobubbles eradicated nearly 100% of phages within 10–15 min, while oxygen nanobubbles preserved phage viability and enhanced their adhesion and uptake in fish tissues. NB-O_2_ increased phage presence in mucus and gills up to fifteenfold and in liver tissues up to 4.75 times compared to untreated controls, without harming the fish.	[89]
AhMtk13a,b	*A. hydrophila* GW3-10	*D. rerio*	Georgia	Myovirus (*Tulanevirus*)	genomic, *in vitro/in vivo*	Zebrafish infection model confirmed its therapeutic efficacy, particularly when administered immediately or prophylactically	[90]
Ahy-Yong1	*A. hydrophila* MDR	*C. aka* Koi	China	*Autographiviridae*	*in vitro/in vivo*	In vivo, untreated diseased fish exhibited 100% mortality, whereas phage groups showed reduced cumulative mortality: 43.3% (post infection treatment), 20% (prophylactic administration), and 30% (simultaneous treatment), also effective biofilm elimination.	[91]
PZL-Ah1 PZL-Ah8	*A. hydrophila*	*C. carpio*	China	Podovirus	*in vitro*	PZL-Ah1 isolated, used broad host range, strong lytic activity, >90% CFU reduction 24 h for strains, improved recovery in vivo when used in cocktail.	[92]
PZL-Ah152	*A. hydrophila*	*C. auratus*	China	Podovirus	*in vitro*	İn vivo infection model using crucian carp; early phagocytic application increased survival rate of fish after infection with *A. hydrophila*.	[93]
φAHBHU12 φAHBHU16 φAHBHU19	*A. hydrophila*	*Pangasius* *buchanani*	India	Podovirus, Siphovirus, Corticoviridae	*in vitro/in vivo*	Phage cocktail effectively prevents *A. hydrophila* infection in *P. buchanani.* Timing and dosage are critical for therapeutic success. Intramuscularly, 1.0 × 10^4^ PFU resulted in a survival rate of 93%, while in immersion models, simultaneous addition of 1.0 × 10^5^–10^6^ PFU mL^−1^ provided 93–100% protection.	[94]
phiA034	*A. hydrophila*, *A. veronii*, *A. jandaei*, *A. bivalvium*	Aquaculture pond water samples	China	Sharonstreetvirus (*Casjensviridae*)	in vitro	PhiA034 exhibited MOI of 1, 20 min. latent period, and burst size of 286 PFU per cell, indicating efficient replication. It showed high stability at 30–60 °C and maintained its infectivity in the pH range of 6–10.	[95]
AHPMCC7	*A. hydrophila* (MTCC 1739)	*Litopenaeus* *vanamei*	India	*Autographiviridae*	*in vitro/in vivo*	In liquid culture experiments, AHPMCC7 showed strong lytic activity by completely lysing *A. hydrophila* within 2 h of incubation. It exhibited a latent period of approximately 10 min. in the growth curve and a burst size of about 275 virions per infected bacterial cell.	[96]
ΦA3	*A. hydrophila*	*Oreochromis* spp.	Vietnam	*Peduoviridae*	*in vitro*	10 phages have shown a relatively broad host range against pathogenic bacterial isolates. Phage ΦA3 has shown highest lytic activity and broadest host spectrum.	[97]
AhFM11	*A. hydrophila* (Hypervirulent)	*Labeo rohita*	India	*Straboviridae*	*in vitro/in vivo*	İn vivo experiments demonstrated high therapeutic efficacy and effective against hypervirulent strains; fish treated with phage showed survival rates of 100% by injection, 95% through immersion, and 93% via oral feeding.	[98]
D6,CF7	*A. hydrophila* MTCC 1739	*C. auratus*, *Labeo rohita*	India	Podovirus	*in vivo*	Oral feed-based trial in rohu with phages D6 (jumbo 259,831 bp) and CF7 (42,439 bp), detecting both phages in kidney tissue at 3.92 ± 0.80 log_10_ PFU/100 mg throughout the 7-day administration period and achieving 65.2% relative percentage survival.	[99]
PZY-Ah	*A. hydrophila*	*C. auratus*	China	not provided	*in vitro/in vivo*	The phage demonstrated strong lytic activity against susceptible *A. hydrophila*, showed favourable biological characteristics, including a relatively short latent period and a high burst size.	[100]
P2, vB_AhydM-H1	*A. hydrophila*	Sewage water	India	Ahphunavirus, Pahsextavirus	*in vitro/in vivo*	P2, vB_AhydM-H1 exhibited narrow host ranges and extended latent periods. Phages remained stable at 40 °C for 1 h, and in pH range 4–10 for 3 h. Genomes of P2 and vB_AhydM-H1 spanned 42,660 bp and 52,614 bp, respectively.	[101]
APT65, AP-Y28, AP-T5 AP-ATCC	*A. hydrophila*	*O. mykiss*	Türkiye	*Straboviridae*	*in vitro/in vivo*	In infection challenge experiments using LD70 dose, oral administration of phage cocktails markedly reduced fish mortality by 32 to 44.8% when fish received feed containing 10^8^ PFU/g phage.	[102]
Ah01, Ah02	*A. hydrophila*	*O. niloticus*	Egypt	Myovirus	*in vitro/in vivo*	Ah01, Ah02 showed high stability at pH values (3–12) and exhibited thermal tolerance up to 84 °C. Latent period of approximately 15 min; burst sizes of 102–209 PFU per infected cell for Ah01-Ah02; polyvirulent activity, infecting several species within *Aeromonas*.	[103]
PZL-Ah152	*A. hydrophila*	*C. auratus*	China	not provided	*in vitro/in vivo*	Hol 46_Lys 17 as a potent agent against resistant *A. hydrophila*, improving carp survival, clearing infections and modulating immunity without toxicity. C-terminal residue role in holin function, fusion’s expanded antibacterial scope, transcriptomic shifts reducing bacterial motility.	[104]
Isolated phage	*A.hydrophila* AH03	*Clarias gariepinus*	Indonesia	not provided	*in vitro/in vivo*	Isolated phages measuring 0.17–0.35 cm reached high titers of approximately 5.68 × 10^9^ PFU/mL. Phage treatment markedly reduced bacterial density compared with untreated controls. The strongest antibacterial effect was observed at MOI 10, 38.7% after 24 h incubation.	[15]
Isolated bacteriophages	*A. hydrophila*	*C. gariepinus*	Indonesia	not provided	*in vitro/in vivo*	Isolated phages markedly inhibited bacterial growth under lab. conditions, reducing density by 50% after 24 h compared to infected controls. In vivo showed that phage improved fish survival relative to the untreated group.	[105]
Z90	*A. hydrophila* 2408	*Anguilla rostrata*	China	Ferozepurvirus (*Chimalliviridae*)	*in vitro/in vivo*	Eel intraperitoneal injection of the phage resulted in a 100% survival rate, indicated outperforming the immersion method, which achieved approximately 70% survival, reduced bacterial loads, and alleviated symptoms like haemorrhaging; immersion was less effective.	[106]
pAEh1	*A. hydrophila*	*Ctenopharyngodon idella*	China	Siphovirus	*in vitro/in vivo*	In infection experiments, carp treated with bacteriophage within 7 days showed a 70% survival rate, while untreated infected fish exhibited high mortality rates. Histopathological results of phage treatment revealed a marked reduction in tissue damage.	[107]
***Aeromonas salmonicida***							
A, B, C, D, E, F, G, H, I, J, K, L, M, N, O, P, Q, R	*A. salmonicida*	*Salmo trutta*	France, UK, Australia, Canada	*Caudoviricetes*	Not stated	In total, 93 *A. salmonicida* isolates were divided into 27 phage sensitivity groups, with the majority of the isolates, total of 57, recovered in only three groups, named 1, 2, and 10.	[108]
HER 110	*A. salmonicida*	*Salvelinus fontinalis*	Canada	not provided	*in vitro/in vivo*	HER110 reduced environmental *A. salmonicida* burden and delayed furunculosis in brook trout. Use of bacteriophages has the potential to prevent infection in 3 days and to minimise the development of phage-resistant strains.	[109]
O, R, B	*A. salmonicida*	*S. salar*, *O. mykiss*	UK	Myovirus	*in vitro/in vivo*	Multiple delivery routes were feasible, but phage did not meaningfully reduce mortality in tested conditions. Phages were used orally, bath treatment, and injection for therapy against *A. salmonicida*. In aquariums with brook trout, concentration (108 CFU/mL) of bacteria decreased by 6 log CFU/mL in 3 days at MOI of 1.	[110]
AS-1	*A. salmonicida*	Water samples	Portugal	Myovirus	Not stated	Phage AS-1 had no detectable impact on the bacterial community structure.	[111]
PAS-1, phiAS4	*A. salmonicida*	*O. mykiss*	South Korea	Myovirus	*in vitro*	Phage PAS-1 showed efficient bacteriolytic activity. In tank experiments, the administration of infected fish exhibited notable protective effects and increased survival rates. It inhibited infection and improved survival and behavioural recovery in trout challenge trials.	[112]
AS-A	*A. salmonicida*	*Solea senegalensis*	Portugal	not provided	*in vitro/in vivo*	AS-A reduced bacterial density and mortality of furunculosis. Results showed that after 6 h of treatment, phage inhibited growth of *bacteria* both in batch cultures and seawater in fish juveniles. Effective biological control in juveniles via immersion (10^10^ PFU/mL).	[113]
SW69-9, L9-6, Riv-10	*A. salmonicida*	Fish	Canada	not provided	Genomic characterisation	Showed substantial genomic diversity among *A. salmonicida* phages, supporting cocktail design. New classification scheme for *A. salmonicida* phages.	[114]
AS-A, AS-D, AS-E	*A. salmonicida*	Aquaculture isolates	Portugal	not provided	*in vitro*	Cocktails improved bacterial control and reduced emergence of resistant mutants under aquaculture conditions. Phage cocktails reduced population of *A. salmonicida* faster than single suspensions.	[115]
AS-szw, AS-yj, AS-zj, AS-sw, AS-gz	*A. salmonicida*	Fish and sewage	China	Myovirus	*in vitro*	Phage adsorption showed that 90% were adsorbed to *A. salmonicida* in first 5 min. Phage AS-gz was highly effective against host at all MOIs tested (0.01, 0.1, 1 and 10), but MOI of 0.01 had best results. Cocktail combining AS-gz and AS-yj was more efficient than other cocktails and phages individually. In vitro investigations into phages are prerequisite to obtain satisfying phage cocktails prior to application in practice.	[116]
AsXd-1	*A. salmonicida*	Waste water of seafood market	China	Siphovirus	Genomic characterisation	Expanded the genomic diversity of *A. salmonicida* phages and identified lysogeny-related genes in AsXd-1.	[117]
ASP-1	*A. salmonicida*	*C.auratus*, trout models	South Korea	not provided	*in vitro/in vivo*	ASP-1 phage was isolated and characterised. Reduced bacterial density and improved survival in infected fish, supporting therapeutic use. Inhibited growth of *A. salmonicida* strains. Phage was stable over wide range of temperatures, pH and salinity. ASP-1 showed 30 min latent period, 16 PFU/infected cell burst size and 40 min rise period.	[118]
T7-Ah (HER212)	*A. salmonicida* subsp. *achromogenes*	Atlantic salmon, Brown trout	Canada	Podovirus	*in vitro*	T7-Ah is the first described podovirus infecting *A. salmonicida* subsp. *achromogenes*, potentially valuable component of phage cocktail strategies targeting the full biological diversity of *A. salmonicida* infections across freshwater and marine aquaculture systems.	[119]
SW69-9	*A. salmonicida*	Sewage	Canada	Myovirus	*in vitro*	Identified LPS and A layer-associated mechanisms influencing phage adsorption and resistance.	[120]
vB_Asa_1 vB_AsM_ZHFZHA, ZHD	*A. salmonicida* subsp. *masoucida*	*Scophthalmus* *maximus*	China	not provided	*in vivo*	*A. salmonicida* subsp. *masoucida* phage isolates sewage, vB_AsM_ZHF exhibited antibacterial effect, based on in vitro experiment. Administered by injection and immersion challenge. Significant reduction in mortality and bacterial burden; decreased inflammatory cytokines (IL-1β and TNF-α).	[121]
HER98, ER110, SW69-9, L9-6, Riv-10	*A. salmonicida subsp. salmonicida*	No direct fish infection model	France, Switzerland, Canada	Myovirus	*in vitro*	Prophage 3 cocktail is specific for *A. salmonicida* strains, may overcome resistance issues and hold promise for preventing furunculosis.	[120]
MQM1	*A.salmonicida* subsp. *salmonicida*	Aquaculture environments	Canada	Podovirus	*in vitro*	Genome size 40,505 bp dsDNA, 53 proteins. Infection cycle 48 min, burst size 71 virions/cell. High host specificity limits large-scale therapeutic application.	[122]
JELG-KS1	*A. salmonicida*	Wastewater samples	Latvia	*Studiervirinae*	*in vitro*	Phage shows efficient replication kinetics and extremely narrow host specificity. Lack of virulence or ARG supports its safety profile. Its limited host range may restrict direct therapeutic use without phage cocktail formulation.	[123]
**Other *Aeromonas* spp.**							
IHQ1	*A. punctata*	Stream water	Pakistan	Myovirus	*in vitro*	Characterisation of phage IHQ1 showed that it was very efficient in lysing *A. punctata*, combined with its outstanding thermal and pH stability, with latent period of approximately 24 min and high burst size (626 PFU/cell), although its narrow host specificity limited broader applicability.	[124]
VTCCBPA5 (BPA5)	*Aeromonas veronii* biovar *sobria*	Pond water	India	Podovirus (T7-like)	*in vitro*	Results demonstrated that BPA5 is a highly host-specific lytic phage with stability across a broad pH 6–10 and temperatures 4–45 °C. Protein analysis revealed a capsid protein (~45 kDa), while genomic and phylogenetic analyses indicated that the phage is distinct from previously described *Aeromonas* podophages.	[125]
pAEv1810 pAEv1812 pAEv1818	*A. veronii*	*Carassius auratus gibelio*	China	*Caudoviricetes*	*in vitro/in vivo*	Phage cocktail markedly inhibited bacterial growth and improved fish survival rates up to 80%, comparable to antibiotic treatment. Importantly, phage administration did not negatively affect growth performance or digestive enzymes.	[126]
phiA034	*A. veronii,* *A. jandaei,* *A. bivalvium*	Specific fish model, Aquatic environment	China	*Caudoviricetes*	*in vitro*	phiA034 possesses infecting 14 strains across four *Aeromonas* spp. Phage exhibited a latent period of 20 min and burst size of 286 PFU. It demonstrated (30–70 °C) and pH (6–10). Genomics revealed 82 ORFs with structural, replication, and lysis functions; phylogenetic classification identified it as a novel species.	[95]
ZPAV-18 ZPAV-25 pAEv1	*A. veronii*	*C. idella*	China	not provided	*in vivo*	Phage ZPAV-25 could adhere to and remain in the mucus of yellow catfish for a week. Fish mucus also could increase *A. veronii* susceptibility to phage infection. High therapeutic efficacy in in vivo fish model. Rapid replication and lytic activity.	[127]
AVP-type phage	*A. veronii*	Wastewater sources	Sri Lanka	Siphovirus	*in vitro/in vivo*	Results demonstrated strong antibacterial activity with rapid bacterial clearance in vitro. İn vivo, phage-treated fish showed higher survival rates compared to untreated controls, along with reduced tissue damage and lower bacterial counts.	[128]
AS-phage	*A. schubertii*	*Channa argus*	China	Myovirus	*in vitro/in vivo*	Effective control of *A. schubertii* infections. Demonstrates strong in vivo therapeutic performance. In total, 83% survival via injection and 100% via immersion, inhibited multiplication in the liver. Genetically safe and environmentally stable.	[129]
AVP-K1	*A. veronii*	Specific fish model	India	Myovirus	*in vitro*	AVP-K1 effectively degraded established biofilms of *A. veronii*, reducing biomass and bacterial viability	[130]
vB_AdhM_DLvB_AdhS_TS3vB_AdhM_TS9 vB_AdhS_M4	*A. dhakensis*	No fish model	Thailand	Myovirus, Siphovirus	*in vitro*	Individual phages and the phage cocktail produced marked bacterial reduction. The phage cocktail plus sub-MIC amoxicillin achieved complete inactivation under in vitro conditions. Phage + antibiotic combination as a practical strategy against resistant *A. dhakensis*.	[131]
vB_AerC_LA93P1 vB_AerC_LA93P2	*A. dhakensis*	*P. hypophthalmus*	Vietnam	*Chaseviridae*	*in vitro*	High burst size and cross-strain lytic activity; useful for cocktail design to limit resistance emergence. Their biological stability matched environmental conditions relevant to striped catfish production. Genomic screening supported biosafety, making them promising biocontrol candidates. A key next step is testing these phages in in vivo striped catfish challenge models.	[132]
pAEv1 (PWJ1_01)	*A. veronii*	*C. idella*	China	not provided	*in vitro/in vivo*	Strong in vitro bactericidal activity with bacterial reduction. ~80% survival in fish model. Supports the use of phage therapy as an effective alternative to antibiotics, but suggests combination strategies for long-term control.	[133]
vB_AceP_Pac	*A. caviae*	Experimental model	China	Myovirus	*in vitro/in vivo*	Phage was isolated and genomically characterised, followed by in vivo infection of model org. and oral phage administration. Parameters such as intestinal inflammation, microbiota composition, and immune response were evaluated.	[134]
AerV_348	*A. veronii* CEMTC 9558	Aquatic isolates	North Kazakhstan	*Caudoviricetes*	*in vitro/in vivo*	AerV_348 is a strictly lytic, host-specific phage with stable physical properties. Its genome expands current knowledge of poorly classified aquatic *Caudoviricetes* phages. Narrow host range may restrict standalone therapeutic use, but it could still be valuable in targeted cocktails or taxonomy-driven phage selection.	[135]

## Data Availability

No new data were created or analysed in this study. Data sharing is not applicable to this article.
